# Multifactorial Influences on Oxygen Consumption Recovery Post-High-Intensity Exercise in Adults: A Case-Control Study

**DOI:** 10.3390/medicina61071213

**Published:** 2025-07-03

**Authors:** Monira I. Aldhahi, Rawan I. Alahmed, Reem H. Almutairi, Haya A. Alqahtani, Hatoon M. Alawad, Rania S. Alkabeer, Leena K. Alqhtani, Mohanad S. Aljubairi

**Affiliations:** 1 Department of Rehabilitation Sciences, College of Health and Rehabilitation Sciences, Princess Nourah bint Abdulrahman University, P.O. Box 84428, Riyadh 11671, Saudi Arabia; rawanalahmed5@gmail.com (R.I.A.); reemalm20@gmail.com (R.H.A.); hayaayed038@gmail.com (H.A.A.); hatoon_alawad@outlook.com (H.M.A.); raniasaleh666@gmail.com (R.S.A.); leenakmaalq@gmail.com (L.K.A.); 2 Lifestyle and Health Research, Health Science Research Center, Princess Nourah bint Abdulrahman University, Riyadh 13412, Saudi Arabia; msaljabiri@pnu.edu.sa

**Keywords:** VO_2_ recovery consumption, body fat percentage, fitness performance fatigability, sleep quality

## Abstract

*Background and Objectives*: Oxygen consumption (VO_2_) recovery plays a critical role in reestablishing homeostasis within multiple physiological processes. This study aimed to assess the differences in the fitness profiles, fatigability, patterns of VO_2_ recovery, and sleep quality among individuals with different body fat percentages. Thus, we evaluated the predictive effects of body fat percentage, CRF, fatigability, and sleep quality on VO_2_ recovery patterns following exercise. *Materials and Methods*: Eighty healthy participants aged 18–52 years were included in this case-control study. The participants were divided into two groups based on body fat percentage: normal-fat (CON; *n* = 40) and high-fat (HFG; *n* = 40) groups. The PSQI questionnaire was used to assess sleep efficiency, and a 10 min walk test was performed to assess fatigability. Both groups underwent a symptom-limited treadmill exercise test to assess VO_2_ using a modified bulk protocol, followed by 6 min of passive recovery. *Results*: The participants in the CON group had a higher mean VO_2_ peak than those in the high-fat-percentage group (*p* = 0.0003). The half-time recovery (T1 and T2) demonstrated higher amounts of VO_2_ in the CON group compared to the HFG group (*p* = 0.0007 and *p* = 0.0005), respectively. Those in the HFG reported greater performance fatigability (*p* = 0.01) and poorer sleep quality compared to the CON group (*p* < 0.001). The multiple linear regression model indicated that a higher recovery amount of VO_2_ was associated with the fat percentage, VO_2_ peak, and fatigability index and explained 72% of the variance (F = 39.58, *p* < 0.001). *Conclusions:* The findings of this study revealed that the participants with higher fat percentages exhibited increased performance fatigability and a reduced peak VO_2_ and reported poor sleep quality compared to the normal group. CPF, body fat, and performance fatigability were associated with VO_2_ recovery after high-intensity exercise. The interplay between body fat, fatigability, sleep quality, and VO_2_ recovery highlights the need for a holistic approach to healthcare.

## 1. Introduction

Exercise recovery plays a crucial role in restoring homeostasis in various physiological systems [1,2] and is a recognized marker of cardiovascular efficiency and fitness [3]. The recovery of oxygen consumption (VO_2_)—also referred to as excess post-exercise oxygen consumption (EPOC)—represents the physiological process by which the body restores pre-exercise oxygen levels [4]. EPOC is a term used to describe the increased amount of oxygen consumed to restore internal balance after exercise, which is closely linked to the overall disruption of the body’s internal equilibrium caused by physical activity [4]. The efficiency of the cardiovascular system in recovering after exercise can be evaluated by examining the kinetics of oxygen recovery [3]. The VO_2_ recovery rate reflects the performance level; hence, a faster VO_2_ recovery suggests better performance [5]. Researchers have aimed to examine the factors and variables that may influence VO_2_ recovery. Previous studies have examined the effects of cardiopulmonary fitness [6], body fat percentage [6], and fatigue [5] on VO_2_ recovery. However, previous studies have not comprehensively examined how multiple factors, including sleep efficiency and perceived fatigability, interact to influence post-exercise VO_2_ recovery in healthy individuals.

Cardiorespiratory fitness (CRF) and fatigability are two factors that have been shown to have different effects on the body [7]. CRF, also referred to as aerobic capacity, is a fundamental element of physical fitness [8] and represents an individual’s ability to efficiently utilize oxygen during sustained physical activities [9]. One of the key measures of CRF is the maximal rate of oxygen consumption (VO_2_ max or peak), which is directly related to physical fitness [10]. In healthy individuals, the consumption of VO_2_ returns rapidly to its normal level following physical activity [11]. It has been shown that the recovery of VO_2_ is strongly associated with a higher aerobic capacity (VO_2_ peak) and increased muscle strength [12]. However, when fatigability occurs, it is predicted to influence performance [13]. It is well known that fatigability is divided into perception and performance [13]. The first refers to subjective changes in sensations experienced by an individual that influence their perceptions of physical and mental exertion during a performance [13]. A previous study indicated that fatigue, along with an increase in training load and inadequate recovery during training sessions, can negatively impact sports performance [14].

Two other important factors are sleep deprivation and high body fat percentage, as both are common issues in Saudi Arabia [15,16]. According to a study conducted in Saudi Arabia, 154 out of 399 participants (38.59%) experienced sleep problems [15]. In addition, Saudi Arabia has a considerably higher rate of obesity than the global average, with approximately 35% of its population affected compared with the global average of 13% [16]. The fat percentage was found to affect VO_2_ recovery [6]. Thus, both of these factors influence VO_2_ recovery. According to a previous study, a decreased percentage of sleep efficiency can lead to an increase in sympathetic activity [17]. It is worth noting that catecholamines are well-known transmitters that enhance LA production [18], which is known to slow the rate of recovery [19].

Obesity is defined as a condition characterized by an excessive accumulation of body fat that surpasses the normal or healthy range for an individual’s age, height, and sex [20]. Sleep and fat indirectly affect CRF, which influences EPOC. It has been found that CRF and EPOC are negatively correlated with each other [19]. The study by Matsuo et al. examined the relationship between cardiorespiratory fitness and excess EPOC in 21 healthy adults who engaged in three cycling protocols: sprint interval training, high-intensity interval aerobic training, and continuous aerobic training. The sessions had matching levels of energy expenditure. The findings showed that individuals with higher fitness levels had lower EPOC, particularly following high-intensity interval aerobic training, suggesting an inverse relationship between fitness and recovery oxygen demand, which is most evident in aerobic-type interval exercise [19]. This finding underscores the complexity of interpreting the relationship between VO_2_ recovery and fitness.

Additionally, a previous study suggested that maintaining CRF is heavily influenced by obtaining a sufficient sleep duration of 6 h [21]. Moreover, research has investigated the link between obesity and CRF, revealing that body mass index (BMI) has an inverse relationship with CRF [22]. Another study identified a connection between high obesity rates and low CRF levels [23].

The importance of the above-mentioned variables lies in their contribution to overall health and well-being [20,24]. Therefore, the objective of this study was to assess the differences in peak oxygen consumption, fatigability, pattern of VO_2_ recovery, and sleep quality among individuals with different body fat percentages. Additionally, the predictive effects of body fat percentage, peak oxygen consumption, fatigability, and sleep quality on VO_2_ recovery after high-intensity exercise were evaluated. VO_2_ recovery has been found to be linked to CRF [6]. However, the interrelationship between sleep efficiency, fatiguability, and body fat percentage as a predictor of VO_2_ recovery after maximal exercise testing to volitional exhaustion has never been investigated. We hypothesized that peak oxygen consumption during high-intensity exercise, the pattern of VO_2_ recovery, sleep quality, and fatigability while walking would differ in the group with a high body fat percentage compared to those with a normal body fat percentage. Thus, we hypothesized that body fat percentage, peak oxygen consumption, fatigability, and sleep quality have a predictive effect on the pattern of VO_2_ recovery after high-intensity exercise. The findings of this study may help clinicians guide interventions and treatment programs to improve performance after exercise and ensure sufficient recovery by considering these factors.

## 2. Materials and Methods

### 2.1. Design and Participants

This case-control study included 80 healthy adult participants aged 18–52 years. Participants were recruited using a convenience sampling strategy via an online survey distributed using Google Forms. The study was designed and reported in accordance with the Strengthening the Reporting of Observational Studies in Epidemiology (STROBE) guidelines for case-control studies to promote transparency, completeness, and methodological rigor [25].

The survey link was distributed through social media platforms. Individuals were contacted and included in the study after confirming that they met the predefined inclusion criteria. The inclusion criteria were as follows: subjects who were able to walk independently, stand upright, and walk on a treadmill and who answered “No” to all the questions in the physical activity readiness questionnaire (PARQ+) [26]. The body fat percentage categories were classified based on the normative values for the general population, considering sex and age, as defined by Gallagher et al. For females, a normal body fat percentage was defined as 21% for ages 20–39 and 23% for ages 40–59, whereas high body fat was classified as 33–39% and 34–40%, respectively. For males, normal body fat percentage was defined as 8% for ages 20–39 and 11% for ages 40–59, whereas high body fat percentage was defined as 20–25% and 22–28%, respectively [27]. Participants were excluded if they had experienced a musculoskeletal injury within the 12 months prior; had a diagnosis of chronic cardiac or pulmonary disease, infectious conditions, psychological disorders, or were pregnant; or were taking medications or had medical conditions that could interfere with their study participation. Individuals who used tobacco products in any form were excluded.

Participants were stratified into two groups based on body fat percentage: normal-body-fat group (*n* = 40) and high-body-fat group (*n* = 40). To ensure adequate statistical power for both between-group comparisons and regression analyses, a priori sample size calculations were performed using G*Power software (version 3.1, Heinrich Heine University, Düsseldorf, Germany). For the independent samples *t*-test, which was used to compare outcomes between the normal- and high-body-fat groups, a medium effect size, alpha level of 0.05, and power of 0.80 indicated a minimum sample size of 64 participants (32 per group). Additionally, for the multiple linear regression analysis involving four predictors, a medium effect size (f^2^ = 0.15), α = 0.05, and power = 0.80 yielded a required sample size of 80 participants.

### 2.2. Ethical Considerations

This study was approved by the Institutional Review Board of Princess Nourah bint Abdulrahman University (HAP-01-R-059; date: 15 February 2024). All participants were fully informed about the study, including its purpose, objectives, procedures, potential risks and benefits, and estimated duration. Written informed consent was obtained from all participants before their involvement. Participation was entirely voluntary, and the participants were informed of their right to withdraw from the study at any time without providing a reason. All personal data and information collected were kept strictly confidential. This study was conducted in accordance with the ethical principles outlined in the Declaration of Helsinki.

### 2.3. Study Procedures

**Figure 1:**
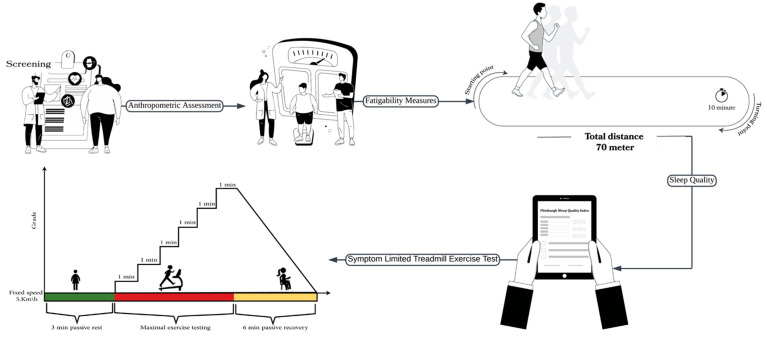
Illustration of the study procedure and the protocol used for the modified Balke treadmill test.

First, the investigators explained the testing procedures in detail to the participants and obtained written consent from all the subjects to participate. The PARQ+ was then administered to all participants, their health histories were recorded, and demographic data, including date of birth, age, gender, education, and contact information, were collected. Figure 1 illustrates the study procedure and protocol used for the modified Balke treadmill test.

#### 2.3.1. Anthropometric Assessment

The weights (kg), heights (m), waist circumferences (cm), HRs, and baseline BPs of all participants were measured. Each participant was required to fast for approximately 3–4 h before measuring body composition. Bioelectrical impedance analysis (BIA) devices (Seca285, Seca, Hamburg, Germany; mBCA 525, Seca, Hamburg, Germany) were used, which is the most popular noninvasive method for body composition assessment. It uses the electrical properties of each tissue to calculate body fat mass (kg and %), fat-free mass (kg and %), BMI (kg)/(m)^2^, and skeletal muscle mass (kg). A low-intensity (approximately 1 mA) alternating electrical current is applied and conducted through the body as part of the BIA measurement process. An analyzer was used to measure BIA [28].

#### 2.3.2. Sleep Quality

Sleep quality was assessed using the Pittsburgh Sleep Quality Index (PSQI), a self-rated questionnaire that evaluates sleep quality and disturbances over a month [29]. The Global PSQI’s internal consistency reliability shows marginal acceptability (Cronbach’s alpha = 0.65) and convergent validity (r = 0.76) [30]. The PSQI consists of 19 items and seven components: sleep quality, latency, duration, disturbances during sleep, medications for sleep, and daytime dysfunction [31]. The global score ranges from 0 to 21, with a score > 5 indicating sleep disturbances [32].

#### 2.3.3. Fatigability Measures

The 10 min walk test (10 MWT) provides a standardized performance tool that allows for the assessment of two fatigability severity measures: perceived fatigability, which is the self-reported change in tiredness, and performance fatigability, which refers to changes in physical performance, offering evidence of concurrent validity for the construct of fatigability in healthy individuals [33]. Participants were asked to walk for 10 min around a pre-measured indoor distance of 70 m per lap. After every 2.5 min, the covered distance was measured and recorded. The primary outcome measure for this test was the total distance covered in 10 min. Performance fatigability was quantified using a fatigability index, calculated as the ratio of the average walking speed over the entire 10 min duration to the walking speed recorded during the initial 2.5 min of the test. In addition, the participants were asked about their pre- and post-perceived exertion using a 7-point scale. Both the pre- and post-perceived exertion scales ranged from 1 to 7, where (1) indicates much more energetic, (2) indicates somewhat more energetic, (3) indicates a little more energetic, (4) indicates neither more tired nor energetic, (5) indicates a little more tired, (6) indicates somewhat more tired, and (7) indicates much more tired. The Performance Fatigability Index was calculated as the ratio of the speed (m/s) over the entire 10 min divided by the speed in the first 2.5 min [33].

#### 2.3.4. Symptom-Limited Treadmill Exercise Test

**Figure 2:**
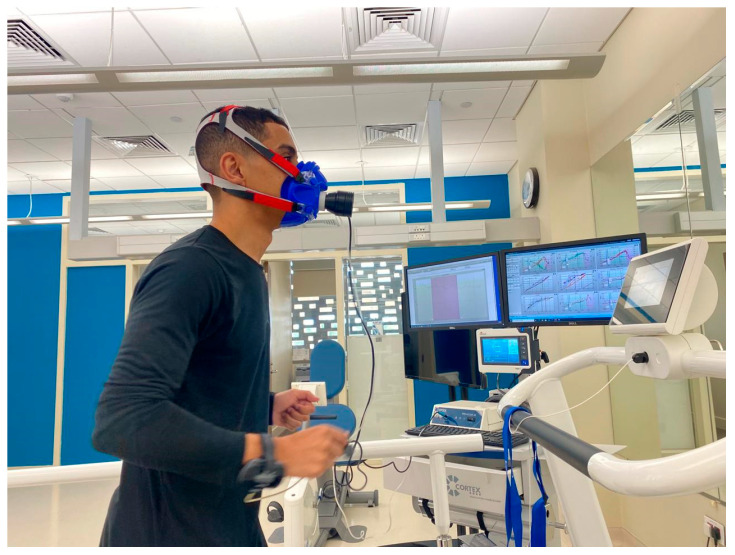
Participant connected to the gas exchange analyzer during the cardiopulmonary exercise test. Image is used with their permission.

The participants then underwent a symptom-limited treadmill exercise test using the modified Balke protocol, as shown in Figure 1. Before the test, the participants’ HRs and BPs were measured. The participants were asked to wear a polar heart rate monitor (Polar H7, Kempele, Finland) to calculate their HRs during the test. A cardiopulmonary exercise test (CPET) was performed using a breath-by-breath gas analyzer device (Cortex Metalyzer 3B, Leipzig, Germany) to determine the amount of oxygen consumption (VO_2_), carbon dioxide (VCO_2_), and minute ventilation (VE), as shown in Figure 2. The test was performed on a treadmill, allowing for adjustments to speed and inclination. The participants were connected to a gas-analyzing metabolic cart (mouthpiece) to determine their oxygen uptake, ventilated VCO_2_, lung volumes, and HR. The choice of the modified Balke protocol is particularly suitable for populations with higher body mass. The literature supports that the modified Balke protocol minimizes initial physical fatigue while progressively increasing exercise load, making it safer and more bearable for overweight or less fit individuals [34,35]. This procedure provides more precise and reliable assessments of cardiorespiratory fitness and exercise tolerance in groups whose high-intensity protocols may result in premature testing due to tiredness or discomfort, thereby limiting the diagnostic value [36].

These tests were conducted on a computerized treadmill, allowing for programmed modifications of both work rate components: speed and inclination. The treadmill test started with 3 min of standing (passive rest), and then the grade was increased by 1% every 1 min at a fixed speed of 3.3 mph (5.3 km/h) throughout the test until the participants reached the level of maximum exertion. After stopping, the participants had a 6 min passive recovery period. Participants were asked to rate their perceived exertion every 2 min. The participants reached their highest peak of effort if at least one of the following criteria was met: HR max > 85%, rate of gas exchange (RER) ≥ 1.05, rate of perceived exertion 6–9 on a scale of 0–20, or failure to elevate the amount of oxygen consumption level with an increased workload of 150 mL.min^−1^ [37].

VO_2_ recovery was calculated based on a 6 min recovery interval during passive recovery (sitting). We considered two periods (T1 and T2). VO_2_ recovery_T1 denotes the half-time of the first 3 min of passive recovery (6 min), whereas VO_2_ recovery_T2 denotes the second half-time of the last 3 min. VO_2_ recovery was calculated to measure the delta (Δ) of change between the peak of VO_2_ and recovery of VO_2_ during the last 1 min of each time period (T1, T2). Supplementary File S1 shows a reference table for abbreviations.

### 2.4. Statistical Analysis

The statistical analysis was performed using Stata version 17 (StataCorp, College Station, TX, USA). Continuous variables are presented as means and standard deviations (SDs), and categorical variables are presented as frequencies and percentages. The normality of the distribution of all outcome variables was checked using the Shapiro–Wilks test. The comparison between the two groups was analyzed using an independent *t*-test for baseline characteristics. Analysis of covariance (ANCOVA) was used to compare groups with different body fat percentages after adjusting for muscle mass. Pearson’s correlation coefficients were used to investigate the correlations between VO_2_ recovery, age, fat percentage, peak oxygen consumption, sleep quality, and fatigability. A forward multiple linear regression analysis was conducted to investigate the association between VO_2_ recovery (dependent variable) and predictive factors (independent variables). Statistical significance was set at an alpha value of <0.05.

## 3. Results

The demographic and physical characteristics of the participants are shown in Table 1. Among the 80 participants, they were categorized into two groups based on body fat percentage: normal (*n* = 40) and high (*n* = 40). Participants in the high-fat-percentage group had a significantly higher BMI (28.03 ± 5.23 kg/m^2^) than those in the normal-fat-percentage group (21.97 ± 2.56 kg/m^2^; *p* < 0.001). Significant differences were also seen in skeletal muscle mass, with the normal-fat-percentage group averaging 21.45 ± 5.45 kg and the high-fat-percentage group averaging 21.84 ± 6.21 kg (*p* < 0.001).

The overall mean age of the participants was 24.53 ± 5.78 years, with no significant age difference between the groups. The male gender accounted for 40% of the total sample, with a higher percentage in the normal-fat-percentage group (47.5%) than in the high-fat-percentage group (32.5%); however, this was not statistically significant. Regarding their educational levels, the majority of the participants (97.5%) held at least a Bachelor’s degree. There was no significant difference in educational level between the groups.

**Figure 3:**
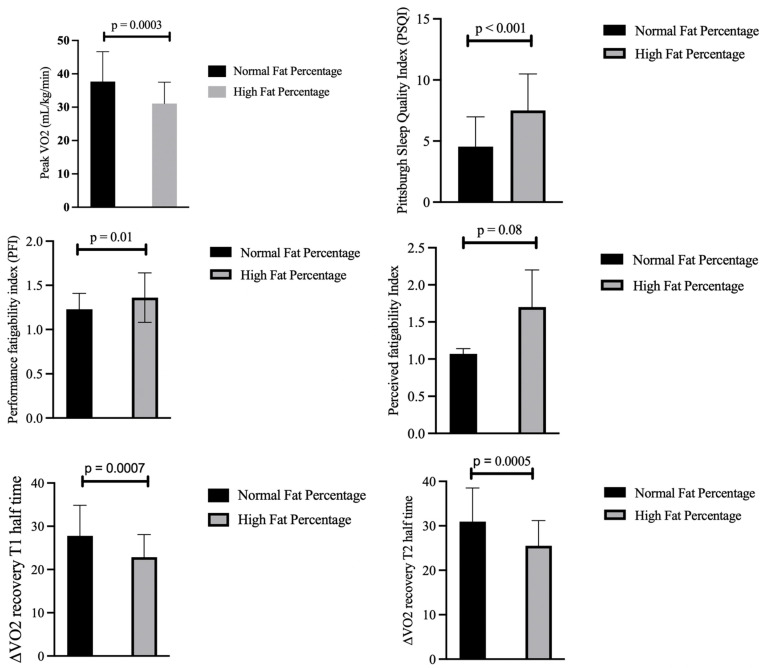
Comparison of fitness profile, sleep quality, and fatigability between individuals with normal and high fat percentages.

Figure 3 shows a comparative analysis between individuals with normal- and high-fat-percentages adjusted for muscle mass using ANCOVA, revealing significant differences in several key parameters. Individuals with a normal fat percentage exhibited high cardiorespiratory fitness, as evidenced by a higher mean VO_2_ peak of 37.69 mL/kg/min (SD = 8.96), compared to 31.10 mL/kg/min (SD = 6.41) in the high-fat-percentage group. Individuals with a normal fat percentage also demonstrated better recovery rates in VO_2_ post-exertion. The half-time recovery during the first 3 min of passive recovery (VO_2_-T1) showed the recovery of a higher amount of VO_2_ (27.77 mL/kg/min) in the normal-fat-percentage group than the high-fat-percentage group (22.86 mL/kg/min). Similarly, the half-time recovery during the second time period of the last 3 min of VO_2_ recovery was significantly higher than that of the high-fat-percentage group (by 5.44 mL/kg/min).

Furthermore, those with higher fat percentages exhibited greater performance fatigability (1.36 (SD = 0.28) versus 1.23 (SD = 0.18) in the normal-fat-percentage group (*p* = 0.01)) and reported poorer sleep quality. However, perceived fatigability did not differ significantly (*p* = 0.08), indicating that subjective fatigue measures may not fully align with physiological markers.

Table 2 presents the Pearson correlation coefficients for the variables measured in this study, including the VO_2_ half-time recovery (T1 and T2), age, body fat percentage, peak VO_2_ performance, Perceived Fatigability Index, and PSQI. These results revealed that body fat percentage and Performance Fatigability Index were strongly negatively correlated with VO_2_ recovery at T2 (r = −0.62, *p* < 0.01; r = −0.3, *p* < 0.01, respectively), highlighting the impact of physical fitness on recovery processes. The correlations with cardiorespiratory fitness (as measured by peak VO_2_) further emphasized the positive relationship with recovery (r = 0.98, *p* < 0.01).

The multiple linear regression model with VO_2_ recovery as the dependent variable, adjusted for muscle mass, is presented in Table 3. Multivariate analyses indicated that a higher recovery amount of VO_2_ was associated with a low fat percentage, high VO_2_ peak, and low Performance Fatigability Index score while walking (*p* < 0.001). Each unit increase in body fat percentage was associated with a 0.27 unit decrease in VO_2_ recovery (β = −0.36, *p* = 0.001). For each unit increase in peak VO_2_, recovery increased by 5.85 units (β = 0.08, *p* < 0.001), and with each unit increase in the PFI, VO_2_ recovery decreased by 41.24 units (β = −0.60, B = −41.24, SE = 17.88, t = −2.31, *p* = 0.025). The final multivariate model explained 72% of the variance in VO_2_ recovery, as measured during the last min of the 6 min recovery period (F = 39.58, *p* < 0.001), indicating a strong model fit (F = 39.58, *p* < 0.001).

## 4. Discussion

This study addresses a critical gap in the literature by examining the complex interplay between cardiorespiratory fitness, performance fatigability, sleep efficiency, body fat percentage, and post-exercise oxygen recovery. Notably, this is the first study to investigate the combined influence of fatigability and sleep efficiency on VO_2_ recovery, a relationship that has not been previously explored. This study revealed significant differences in VO_2_ recovery following high-intensity exercise between individuals with high and normal body fat percentages. Furthermore, the results demonstrated that VO_2_ recovery was significantly associated with body fat percentage, sleep quality, performance fatigability, and VO_2_ peak, an established indicator of cardiopulmonary fitness, across individuals with varying body compositions.

Furthermore, the results of the current study indicated significant associations between fitness level and sleep, as well as between performance fatigability and BFP. These findings are consistent with previous research demonstrating an inverse relationship between higher body fat and lower fitness levels [38]. However, perceived fatigability, which reflects the subjective experience of fatigue, did not significantly differ between groups with different fat percentages. This apparent discrepancy implies that while objective measures of performance fatigability (e.g., a decline in walking speed) worsened with increased body fat, participants did not necessarily perceive themselves as more fatigued. Future studies should investigate the psychological and physiological mechanisms underlying this dissociation, as it may have implications for how fatigue is assessed and managed in clinical and athletic populations.

Additionally, the present study found a relationship between sleep efficiency and body fat percentage. Specifically, the results show that a higher fat % was significantly associated with lower sleep quality and sleep efficiency. These results are consistent with a previous study [39]; however, BMI was measured using DEXA scans and sleep was measured using wrist-worn accelerometers and sleep diaries over a 7-day period.

The findings of our study indicate that there are significant differences in VO_2_ recovery following high-intensity exercise between individuals with high and normal body fat percentages. This finding aligns with the results of a study that examined the relationship between VO_2_ recovery, heart rate, body composition, and aerobic fitness using the 30 s Wingate test on 14 professional cyclists and found that individuals with higher levels of body fat had delayed heart rate recovery and higher EPOC [6]. However, it is important to note that their protocol substantially differed from ours. The use of a short-duration, high-intensity cycling task may not adequately reflect the physiological demands of daily activities. In contrast, our study utilized a treadmill-based protocol that more closely simulated real-life functional movements, such as walking. Furthermore, VO_2_ recovery was systematically measured at 3 min intervals following exercise, providing a more comprehensive profile of post-exercise recovery dynamics. Notably, Wong and Harber [40] reported contrasting results. Their results indicated that obese individuals demonstrated lower EPOC and a higher respiratory exchange ratio (RER), which differs from the outcomes of the present study. Several factors may account for this discrepancy, including the small sample size and methodological differences. Specifically, their study employed a submaximal cycling protocol at >60% of VO_2_ max for 30 min, along with an extended recovery monitoring period ranging from 30 to 130 min post-exercise. These variations in exercise intensity, modality, and recovery duration may have influenced the physiological responses measured and may have contributed to the differences in results.

Notably, obese participants exhibited prolonged VO_2_ recovery. In our study, we hypothesized that sleep efficiency influences VO_2_ recovery. However, the findings revealed no significant association between VO_2_ recovery and sleep quality in individuals with different body compositions. This result contrasts with that of the prior literature. For instance, a study involving 50 healthcare professionals compared heart rate (HR) recovery between nightshift and dayshift groups, reporting delayed HR recovery in the nightshift group [41]. From a physiological perspective, prior research has indicating that a slower HR recovery is associated with a prolonged duration of EPOC [11]. Furthermore, another study found that both sleep quality and quantity were associated with declines in VO_2_ max among individuals with poor sleep [21]. Given the established link between VO_2_ max and VO_2_ recovery [13], it was reasonable to speculate that sleep disturbances might indirectly impair VO_2_ recovery via reductions in VO_2_ max. However, our findings do not support this hypothesis, as variations in sleep efficiency did not significantly influence VO_2_ recovery in the present sample. Despite the well-established link between sleep quality, sympathetic activity, and cardiorespiratory responses [21,41], our findings suggest that sleep quality may play a more indirect or context-dependent role in influencing recovery, particularly in healthy individuals. We recommend that future studies incorporate objective sleep metrics—such as actigraphy or polysomnography—and include more diverse samples with varying sleep profiles to clarify this relationship.

In the current study, cardiorespiratory fitness was significantly associated with VO_2_ recovery in individuals with different body compositions. This is consistent with prior evidence suggesting that variations in fitness levels may contribute to differences in the magnitude and duration of EPOC [42]. Moreover, another study demonstrated a negative correlation between EPOC and cardiorespiratory fitness during sprint interval training and high-intensity interval aerobic exercise [19], supporting the premise that higher fitness levels are associated with faster recovery and lower post-exercise oxygen demand. However, the present findings contrast with those of several earlier studies that reported no significant association between cardiorespiratory fitness and VO_2_ recovery [6,8,43]. A study using a cycling ergometer protocol found that fitness level did not influence VO_2_ recovery [8]. Similarly, another study assessed the relationship between VO_2_ max and relative EPOC during both moderate-intensity continuous exercise and high-intensity interval training. The authors reported no significant association between VO_2_ max and EPOC during either exercise [43]. These discrepancies may be attributed to methodological differences, including the use of cycling ergometers in the aforementioned studies and the limitations of small sample sizes.

The last factor is fatigability, which was found to be associated with VO_2_ recovery among individuals with different body compositions in the present study. Few studies have assessed the effect of fatigue on VO_2_ recovery. A prior study found that Borg’s rating of perceived exertion was moderately and significantly associated with the EPOC time constant and HR recovery at 1 min. However, the RPE was not significantly correlated with the magnitude of EPOC [5], which is consistent with the findings of the current study. However, they only investigated one aspect of fatigability: perceived fatigability during submaximal treadmill exercise [5]. In contrast, another study found no association between locomotor muscle fatigue and the VO_2_ slow component during aerobic exercise, which contradicts our results. This discrepancy may be explained by our methodological differences. Specifically, their fatigue protocol involved a 33 min intermittent drop-jump task (100 repetitions) followed by a 6 min high-intensity cycling bout to assess VO_2_ kinetics during the latter. Additionally, their sample was limited to male participants and a small sample size [44]. Importantly, the present study assessed both performance and perceived fatigability, offering a more comprehensive evaluation of fatigue and its relationship with VO_2_ recovery.

The present study has several limitations. First, the sample included participants aged 18–52 years, representing a limited subset of the general population. As such, the findings may not be generalizable to younger adolescents, older adults, or elderly populations, whose physiological responses to exercise and recovery may differ significantly. Second, body composition was assessed using bioelectrical impedance analysis. While BIA has been validated in previous studies and shown to demonstrate strong agreement with dual-energy X-ray absorptiometry, which is considered the gold standard, it remains a less precise method and is susceptible to fluctuations based on hydration status and other individual factors. Given that our study involved participants with a wide range of body fat percentages, this limitation may have introduced variability in fat mass estimates and, consequently, in their associations with VO_2_ recovery.

Furthermore, this study did not account for differences in terms of gender distribution, training history, involvement in sports, or physiological responses between males and females. Gender-specific factors—such as hormones and cardiovascular function—may influence both VO_2_ recovery and fatigue profiles. Therefore, the lack of a gender-stratified analysis may have masked important subgroup variations. Future research should aim to include a broader age range, employ gold-standard tools such as DXA for body composition assessment, and examine potential gender-based differences in VO_2_ recovery. Addressing these factors may yield more nuanced insights into the complex interplay between physiological and behavioral health parameters.

## 5. Conclusions

The findings of this study demonstrate that VO_2_ recovery following maximal exercise testing differs significantly between individuals with normal and high body fat percentages. Cardiorespiratory fitness and performance fatigability were associated with VO_2_ recovery, whereas perceived fatigability and sleep quality were not. These differences suggest that individuals with elevated body fat may exhibit altered recovery patterns, potentially affecting their exercise tolerance and overall cardiovascular health. Importantly, this study contributes to addressing a gap in the literature by identifying specific physiological and behavioral factors that influence VO_2_ recovery dynamics. The observed variability in recovery patterns among individuals with different body compositions highlights the importance of personalized approaches to exercise prescription and recovery. Given the critical role of VO_2_ recovery in maintaining physiological homeostasis, clinicians and practitioners should consider key predictors, namely body fat percentage, CRF, and performance fatigability, when designing intervention programs aimed at optimizing recovery and enhancing performance outcomes. Clinicians and practitioners are recommended to assess CRF and body composition in the design of training or rehabilitation programs, especially for individuals with high adiposity. The individualization of recovery periods and training intensities to accommodate the slower recovery in this group has the potential to prevent overtraining and promote adherence. In addition, enhancing CRF and reducing body fat through individualized interventions can maximize the effectiveness of health and fitness programs in relation to performance and long-term health outcomes.

## Figures and Tables

**Table 1:** Sociodemographic and physical characteristic of the participants.

Variable	All *n* = 80 Mean ± SD	Fat Percentage		*p*-Value
		Normal *n* = 40 Mean ± SD	High *n* = 40 Mean ± SD	
Age (years)	24.53 ± 5.78	24.67 ± 5.73	24.4 ± 5.91	0.8
Weight (kg)	68.24 ± 16.53	60.61 ± 9.83	75.87 ± 18.38	<0.001 *
Height (cm)	164 ± 8.1	165 ± 8.3	163 ± 7.9	0.29
BMI (kg/m^2^)	25 ± 5.1	21.97 ± 2.56	28.03 ± 5.23	<0.001 *
Fat-free mass (kg)	46.90 ± 10.16	46.56 ± 9.41	47.2 ± 10.96	0.76
Skeletal muscle mass (kg)	21.64 ± 5.81	21.45 ± 5.45	21.84 ± 6.21	<0.001 *
Absolute fat (kg)	21.34 ± 10.23	14.05 ± 4.22	28.623 ± 9.25	<0.001 *
Fat percentage	30.17 ± 9.47	22.86 ± 7.02	37.47 ± 4.81	<0.001 *
Male gender, *n* (%)	32(40)	19 (47.50)	13 (32.50)	9.17
Educational level, *n* (%)				
Bachelor’s degree	78 (97.5)	40 (100)	38 (95)	0.4
Post-graduate degree	2 (2.5)	0	2 (5)	
Baseline VO_2_ (mL/kg/min)	3.5 ± 0.2	4 ± 0.4	3.5 ± 0.3	0.8

* Statistically significant (*p* < 0.05). Abbreviations: BMI: body mass index; VO_2_: oxygen consumption.

**Table 2:** A matrix of the Pearson correlations between VO_2_ recovery, fat percentage, fitness, sleep quality, and fatigability.

Variables		Correlation Coefficient						
	1	2	3	4	5	6	7	8
VO_2_ recovery T1 half-time (T1)	1							
VO_2_ recovery T2 half-time (T2)	0.97 *	1						
Age	−0.07	−0.04	1					
Fat %	−0.62 **	-0.62 **	0.06	1				
Peak VO_2_ (mL/kg/min)	0.98 **	0.98 **	−0.07	−0.64 **	1			
PFI	−0.31 **	−0.31 **	−0.07	0.22 *	−0.31 **	1		
Perceived Fatigability Index	−0.161	−0.15	−0.14	0.12	−0.16	0.22 *	1	
PSQI	−0.08	−0.11	0.09	0.33 **	−0.10	0.05	0.11	1

* Denotes correlation is significant at *p* value less than 0.05; ** denotes correlation is significant at *p* value less than 0.01. Abbreviation: VO_2_: oxygen consumption; PFI: Performance Fatigability Index; T1: half-time recovery; T2: half-time recovery; PSQI: The Pittsburgh Sleep Quality Index.

**Table 3:** Multiple linear regression analysis with VO_2_ recovery as dependent variable.

Model	Predictors ^a^	Coefficients							R^2^	VIF
		β	B	SE	T	*p*-Value	95% CI			
							Lower	Upper		
Model *	(Constant)	-	27.93	3.47	8.04	<0.001	21.01	34.86	0.72	-
	Fat %	−0.36	−0.27	0.08	−3.33	0.001	−0.44	−0.11		1.08
	Peak VO_2_ (mL/kg/min)	0.08	5.85	0.68	8.53	<0.001	4.48	7.21		1.14
	PFI	−0.60	−41.24	17.88	−2.31	0.025	−76.84	−5.63		0.8
	Sleep quality	−1.42	0.13	0.16	0.83	0.40	−0.18	0.45		0.9
	F	F (5, 74) = 39.58								
	*F*-value	<0.001								

Abbreviations: B: unstandardized beta regression coefficient; β: standardized beta. ^a^ Predictors: fat percentage (Fat%); peak oxygen consumption (VO_2_); Performance Fatigability Index (PFI); VIF: variance inflation factor. * Regression model was adjusted for muscle mass, and significance level was set at *p* < 0.05.

## Data Availability

Data are available for research purposes from the corresponding author upon reasonable request.

## Supplementary Materials

The following supporting information can be downloaded at: https://www.mdpi.com/article/10.3390/medicina61071213/s1, Supplementary File S1. Acronym Reference Table.

## Abbreviations

The following abbreviations are used in this manuscript:

BIA	Bioelectrical impedance analysis
VO_2peak_	Peak oxygen consumption
T	Half-time
EPOC	Excess post-exercise oxygen consumption
CRF	Cardiorespiratory fitness
VO_2_ max	Maximal rate of oxygen consumption
BMI	Body mass index
HRR	Heart rate recovery
RER	Respiratory exchange ratio
PARQ+	Physical Activity Readiness Questionnaire
PSQI	Pittsburgh Sleep Quality Index
10 MWT	10 min walk test
CPET	Cardiopulmonary exercise test
