# Supplementary material for: Multifactorial Influences on Oxygen Consumption Recovery Post-High-Intensity Exercise in Adults: A Case-Control Study
Source: Medicina (Kaunas). 2025 Jul 3;61(7):1213. doi: 10.3390/medicina61071213 (PMC12298553; doi:10.3390/medicina61071213)
Supplement: Supplementary file 1 [file medicina-61-01213-s001.zip › medicina-3734791-supplementary.pdf]

Supplementary File 1. Acronym Reference Table

Glossary of Acronyms and Key Terms Used in the Study

Acronym	Full Term	Explanation
**VO_2**	Oxygen Consumption	The volume of oxygen consumed per minute; a measure of aerobic metabolism.
**VO_2 peak**	Peak Oxygen Consumption	The highest VO_2 value reached during a graded exercise test; indicator of cardiorespiratory fitness.
**EPOC**	Excess Post-Exercise Oxygen Consumption	Additional oxygen consumed after exercise to restore homeostasis.
**CRF**	Cardiorespiratory Fitness	The efficiency of the heart and lungs to supply oxygen during physical activity.
**PFI**	Performance Fatigability Index	Ratio of average walking speed to initial walking speed during the 10-min walk test.
**T1**	Half-Time Recovery (First 3 Min)	VO_2 recovery rate during the first half of the 6-minute post-exercise period.
**T2**	Half-Time Recovery (Last 3 Min)	VO_2 recovery rate during the second half of the 6-minute post-exercise period.
**PSQI**	Pittsburgh Sleep Quality Index	A self-reported questionnaire used to assess sleep quality and disturbances.
**RER**	Respiratory Exchange Ratio	The ratio of carbon dioxide output to oxygen uptake during exercise.
**BIA**	Bioelectrical Impedance Analysis	A method for estimating body composition, particularly fat mass and lean mass.
